# Fuzzy Inference System Approach for Locating Series, Shunt, and Simultaneous Series-Shunt Faults in Double Circuit Transmission Lines

**DOI:** 10.1155/2015/620360

**Published:** 2015-08-30

**Authors:** Aleena Swetapadma, Anamika Yadav

**Affiliations:** Department of Electrical Engineering, National Institute of Technology, Raipur 492010, India

## Abstract

Many schemes are reported for shunt fault location estimation, but fault location estimation of series or open conductor faults has not been dealt with so far. The existing numerical relays only detect the open conductor (series) fault and give the indication of the faulty phase(s), but they are unable to locate the series fault. The repair crew needs to patrol the complete line to find the location of series fault. In this paper fuzzy based fault detection/classification and location schemes in time domain are proposed for both series faults, shunt faults, and simultaneous series and shunt faults. The fault simulation studies and fault location algorithm have been developed using Matlab/Simulink. Synchronized phasors of voltage and current signals of both the ends of the line have been used as input to the proposed fuzzy based fault location scheme. Percentage of error in location of series fault is within 1% and shunt fault is 5% for all the tested fault cases. Validation of percentage of error in location estimation is done using Chi square test with both 1% and 5% level of significance.

## 1. Introduction

In general, the transmission line faults are categorized into series faults and shunt faults. Unlike the shunt faults, which are characterized by substantial increase in current flow, the low magnitude of current following a series fault makes it difficult to be located by conventional approaches based on calculation of impedance and using fundamental component of current and voltage. Series fault is defined as a fault for which the impedances of the three phases are not equal, which is usually caused by the interruption of one or two phases. Series faults in EHV lines may occur due to broken conductor or a circuit breaker malfunction in one or more phases. The broken conductor leads to unbalance and flow of asymmetrical current arising because of the open conductor coming in series with the effected lines. As per field studies, a series fault may occur due to one of the following reasons:

- Broken conductor(s) due to storm, falling of trees.
- When poles of the circuit breakers fails to open.
- Opening of jumpers at tension tower (angle locations) due to accident and storms.
- Mechanical failure of jumpers.
- Burning of jumper cones due to local heating at joints because of loose contacts/high contact resistance during prolonged operation.

Although series fault is not dangerous to the system, the operation of the load connected is hampered. The numerical distance relays, which are widely used for protection of transmission lines, only give an alarm that particular phase(s) is/are open, but no trip command is issued to the circuit breaker. Further the distance relays are unable to locate the open conductor (series) fault. Faults in transmission lines affect the power flow and reduce the reliability of transmission system. Fault location estimation is an important task in transmission system for carrying out maintenance work to improve power flow reliability and reduce repairing expenses.

Some research has been done to detect the open conductor/series fault. Open phase conductor detector system is described in [1] consisting of transmitters and receiver where transmitter(s) detects the open phase conductor by monitoring the phase conductor voltage using redundant inputs. Carrier communication is used for open conductor detection in [2]. Open conductor fault calculation in four parallel transmission lines using twelve-sequence component methods is discussed in [3]. ANN based techniques are used for enhancement of distance relay performance against open conductor in HV transmission lines in [4]. However these schemes [1–4] are unable to find the location of series/open conductor fault.

There are many schemes reported for shunt fault location estimation. Phasor measurements units (PMU) are used by many researchers nowadays for finding the location of shunt faults in transmission lines [5–17]. Phasor measurement fault location algorithms can be categorized as synchronized [5–9] and unsynchronized [10–14]. Synchronized measurement can be achieved using global positioning system (GPS) and high speed broad band communication system. GPS has a remote telemetry unit which provides the synchronized data through transmission control center [5]. Fault analysis functions, such as fault detection, classification, and location, are implemented for a transmission line using synchronized samples from two ends of a line in [6]. Traveling-wave based fault location techniques for transmission grids using synchronized voltage measurements are proposed in [7]. Synchronized current measurement from both ends and voltage measurement from one end are used for fault location estimation in [8]. In [9] only half cycle of the postfault synchronized voltage and current samples from both ends of the line are taken to estimate location of fault. Fault locations are also estimated using two-end unsynchronized phasor measurement in series-compensated lines [10–12]. Shunt fault location estimation algorithms can be categorized in terms of using one-end data [13–17] and two-end data [6, 7, 9–12]. Phase coordination approach using one-end data for fault location estimation is proposed in [13, 15, 16]. Differential equation approach using one-end data is proposed in [14, 17]. Fault distance is also estimated using modal theory by taking two-terminal data [11]. It is noteworthy to mention here that these schemes [5–17] are not applicable for series/open conductor fault location estimation task.

Fault location algorithms for shunt faults are reported by researchers using different soft computing techniques like artificial neural network (ANN) [18–21], fuzzy [22] and adaptive neurofuzzy inference system (ANFIS) [23], SVM [24], and so forth. Among all the soft computing techniques, fuzzy inference system is used mostly in engineering applications, for example, fault classification [25], due to its easy implementation and less computation work to get accurate results unlike other training based soft computing methods. Moreover there is a chance that simultaneous series and shunt faults may occur in the transmission line as discussed in [26, 27] which can lead to incorrect operation of relay. Digital distance relaying scheme which takes care of a simultaneous open conductor and ground fault occurring coincidently on the same phase at the same point on a series-compensated double circuit line is proposed in [28]. But the scheme treats the simultaneous open conductor and ground fault as single line to ground fault.

Hence, it can be concluded that, hitherto, none of the earlier reported papers [1–28] can locate both series and shunt faults and simultaneous series-shunt faults. In this paper, a method is proposed using synchronized phasors and fuzzy logic to classify the fault and estimate fault location of series faults, shunt faults, and simultaneous series and shunt faults. The proposed fuzzy based method works in three stages. In the first stage, the current and voltage signals obtained from both ends of the line are preprocessed to calculate the fundamental components and zero-sequence component of current signals. Thereafter, two fuzzy modules for fault classification have been designed to discriminate the type of fault, that is, whether series fault or shunt fault or simultaneous series and shunt fault has occurred. Further, according to the type of fault, a particular fuzzy module for fault location of series or shunt or simultaneous series or shunt will be activated which finds the location of fault in kilometers from the relaying point.

## 2. Fuzzy Inference System (FIS) and Its Application

Fuzzy inference system is chosen to locate the faults in transmission lines in this work because it is easy to implement and it does not require training module to produce outputs. Due to less computation work than other soft computing techniques fuzzy system is chosen. Fuzzy inference system deals with fuzzy logic which starts with the concept of a fuzzy set. A fuzzy set is a set without a crisp, clearly defined boundary. It can contain elements with only a partial degree of membership. A fuzzy set can be defined by the following expression:

(1) $$\begin{array}{c} A=\left\{\left(x,\mu_{D}\left(x\right)\right)I_{x}\in X,\;\;\mu_{D}\left(x\right)\in \left[0,1\right]\right\}, \end{array}$$

where  *X* represents the universal set, *x* is an element of *X*, *D* is a fuzzy subset in *X*, and *μ*_*D*_(*x*) is the membership function of fuzzy set *D*.

FIS chosen to be used here is “Mamdani” type because it expects the output membership functions to be fuzzy sets. As fuzzy logic used here is to estimate the location of fault which is not a fixed value, so it is better to use Mamdani method than to use Sugeno method. Membership functions are designed with various membership functions like Gauss, triangular, trapezoidal, and sigmoid functions and so forth. In this work input and output are designed with triangular member function because it has lowest error in location. The triangular membership function is a function of a vector, *x*, and depends on three scalar parameters, *a*, *b*, and *c*, as given by (2) or (3). Consider the following:

(2) $$\begin{array}{c} f\left(x;a,b,c\right)=\left\{\begin{array}{cc} 0, & x\leq a\\ \frac{x-a}{b-a} & a\leq x\leq b\\ \frac{c-x}{c-b}, & b\leq x\leq c\\ 0, & c\leq x \end{array}\right. \end{array}$$

or

(3) $$\begin{array}{c} f\left(x;a,b,c\right)=max\left(\min\left(\frac{x-a}{b-a},\frac{c-x}{c-b}\right),0\right). \end{array}$$

Fuzzy sets and fuzzy operators are the subjects and verbs of fuzzy logic. If-then rule statements are used to formulate the conditional statements that comprise fuzzy logic. A single fuzzy if-then rule assumes the form as shown in

(4) $$\begin{array}{c} \text{IF }x\text{ is }A\text{ THEN }y\text{ is }B, \end{array}$$

where *A* and *B* are linguistic values defined by fuzzy sets on the ranges *X* and *Y*, respectively. From the inputs impedance values their membership values are obtained. This process is called “input fuzzification.” From the consequent of each rule (a fuzzy set) and the antecedent value obtained a fuzzy implication operator is applied to obtain a new fuzzy set. Implication method used here is “minimum” which truncates the consequent's membership function and the product which scales it. Then it combines the outputs obtained for each rule into a single fuzzy set, using a fuzzy aggregation operator which is “maximum” in this case. The fuzzy set is then transformed into a single numerical value. Defuzzification method used here is the “centroid” method which returns the centre of the area under the fuzzy set. Center of gravity method is a grade weighted by the areas under the aggregated output functions. The centroid defuzzification method can be given as in

(5) $$\begin{array}{c} Z^{*}=\frac{\int \mu_{c}\left(z\right)z\;dz}{\int \mu_{c}\left(z\right)dz}, \end{array}$$

where ∫*μ*_*c*_(*z*)*dz* ≠ 0 for all *μ*_*i*_.

By following all these steps described above fuzzy module is designed for fault classification and location estimation module. Detailed fuzzy design of the proposed fault location schemes for series faults, shunt faults, and simultaneous series and shunt faults will be described in next section.

## 3. Proposed Fuzzy Based Series, Shunt, and Simultaneous Series and Shunt Faults Classification and Location Estimation

**Figure 1:**
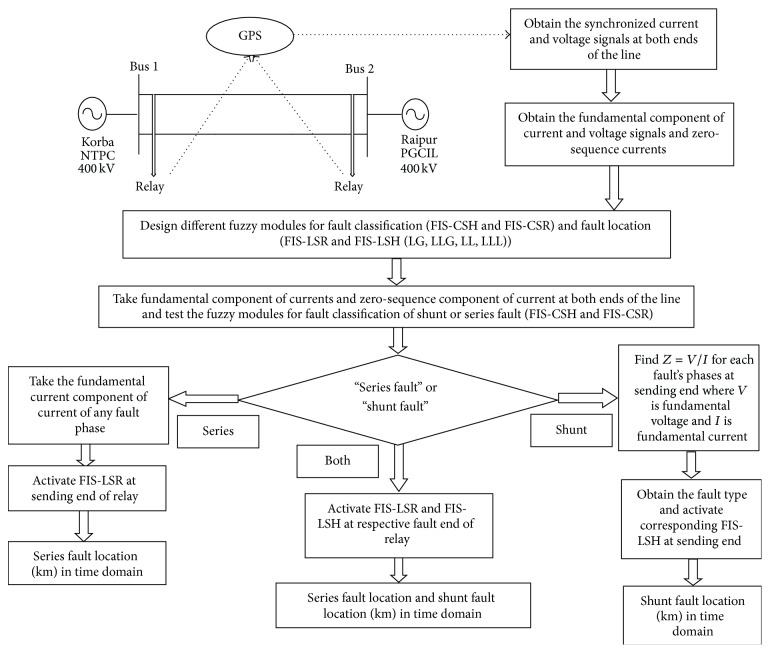
Flow chart of proposed method.

Proposed method uses synchronized phasor measurements and fuzzy inference system to estimate the fault location. Steps followed in the proposed method are described in Figure 1 for the estimation of fault location and are described in following subsections.

### 3.1. Power System Network

**Figure 2:**
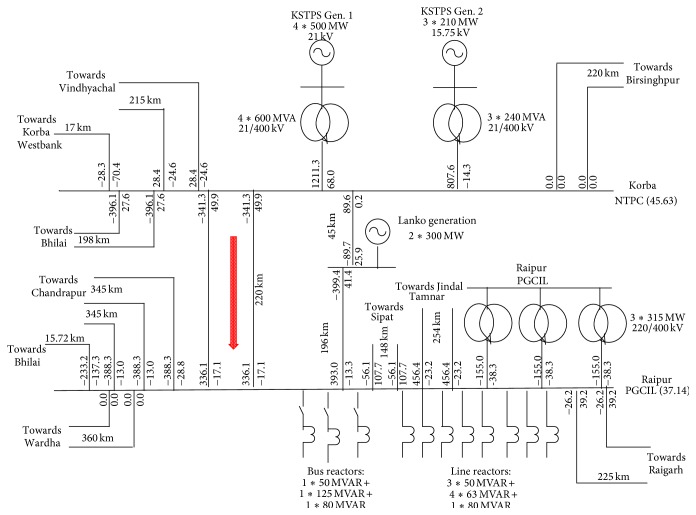
Existing 400 kV transmission line between Korba NTPC to Raipur PGCIL in Chhattisgarh.

The utility electrical power plant system selected for modelling is the existing 400 kV transmission line between Korba NTPC to Raipur PGCIL in Chhattisgarh state. Length of the transmission line is 220 km between Korba to Raipur as shown in Figure 2. Power transfer through the double circuit line is 341 MW. Synchronized phasors of currents and voltages are preprocessed using Discrete Fourier Transform (DFT) and sequence analyzer. Fundamental component of current and voltages is obtained using DFT. Zero-sequence currents are obtained using the sequence analyzer in order to determine whether ground is involved in the fault loop or not.

### 3.2. Design of Fuzzy Module for Classification of Series Fault (FIS-CSR) and Shunt Fault (FIS-CSH)

For classification of type of faults, the fundamental components and zero-sequence components of currents of both ends are taken. Two different FIS modules are designed, one for classification of series fault (FIS-CSR) and the other for classification of shunt fault (FIS-CSH). In this present study Mamdani type FIS has been used because its outputs are in fuzzy sets.

#### 3.2.1. Fuzzy Module for Classification of Series Fault (FIS-CSR)

**Figure 3:**
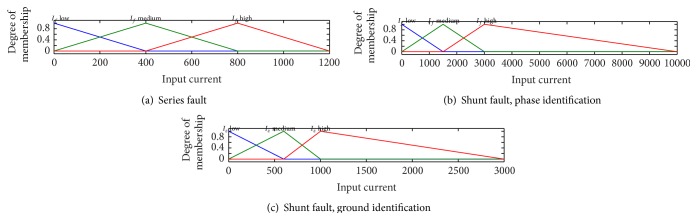
Degree of member function for (a) series fault, (b) shunt fault phase identification, and (c) shunt fault ground identification.

A single FIS module has been designed for detecting the presence of fault in a particular phase. The same FIS has been used for the other two phases. Each phase FIS module takes its fundamental phase current as input and provides single output representing the presence of fault in that phase by trip high (TH) or trip low (TL) for no fault condition. The fundamental components of three phase currents of both circuits measured at both ends of the line are used as input for fault classification. Fundamental component of current *I*_*f*_ is set to certain range which corresponds to fault or no fault in each phase. Three ranges of *I*_*f*_ are selected using triangular member function, that is, *I*_*f*_ low, *I*_*f*_ medium, and *I*_*f*_ high. The output trip logic also contains two ranges of triangular member function, that is, trip low (TL) (0) and trip high (TH) (1). The degree of membership functions for input phase fundamental current is shown in Figure 3(a) for series faults. FIS-CSR has six outputs corresponding to the three phases of the parallel lines (double circuit lines). The rules designed for faulty phase identification and fault classification are as follows:

(1) If fundamental phase current is *I*_*f*_ low or *I*_*f*_ medium then trip is TH.
(2) If fundamental phase current is *I*_*f*_ high then trip is TL.

Fuzzy inference system for fault classification of series fault (FIS-CSR) takes the fundamental current of each phase as input and produces the state of each phase (whether faulty or not) as output.

#### 3.2.2. Fuzzy Module for Classification of Shunt Fault (FIS-CSH)

The shunt faults are classified into phase to ground (LG), double phase to ground (LLG), phase to phase (LL), and three-phase (LLL) faults. As discussed in Section 2, for faulty phase identification, the fundamental components of three phase currents are taken as input to FIS-CSH for classification of phases. Further, for ground identification, separate FIS module has been designed which takes the zero-sequence current signals of the two circuits 1 and 2 as input. Each input's signals are distributed in three ranges with triangular member function, that is, low, medium, and high. There are six outputs for faulty phase identification corresponding to the three phases of circuit 1: A1, B1, and C1 and A2, B2, and C2 of circuit 2 which becomes high (1) in case of fault and otherwise remains low (0). The degree of membership functions for input current is shown in Figure 3(b) for shunt fault phase identification and in Figure 3(c) for ground identification. The rules designed for faulty phase identification are as follows:

(1) If fundamental phase current is *I*_*f*_ low or *I*_*f*_ medium then trip is TL.
(2) If fundamental phase current is *I*_*f*_ high then trip is TH.

Further the rules used for ground identification are as follows:

(1) If zero-sequence current is *I*_*z*_ low or *I*_*z*_ medium then trip is TL.
(2) If zero-sequence current is *I*_*z*_ high then trip is TH.

### 3.3. Design of Fuzzy Module for Fault Location

Once the fault is detected and its type is identified, then the next task of protective relaying scheme is to estimate the fault location from the relaying point. In this study, two separate FIS modules have been designed for series fault (FIS-LSR) and shunt fault (FIS-LSH). Based on type of fault that has occurred in the monitored transmission line, that is, whether series fault or shunt fault or simultaneous series-shunt fault, the corresponding fuzzy module for fault location will be activated which estimates the location of fault.

#### 3.3.1. Fuzzy Module for Series Fault Location (FIS-LSR)

**Figure 4:**
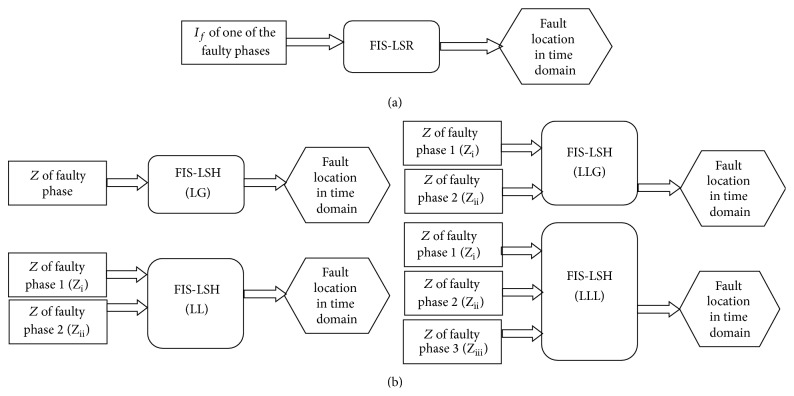
Fuzzy modules: (a) series FIS-LSR and (b) shunt FIS-LSH for fault location.

During series fault, the fundamental component of current signals is the same in all the faulted phases for fault at a particular location (either single phase open fault or multiple phase open faults). Hence only one faulty phase fundamental current is taken as input for design of fuzzy location module for series faults. The block diagram of fuzzy module designed for fault location estimation of series faults (FIS-LSR) is shown in Figure 4(a).

Fundamental components of current values are divided into ranges like *I*_1_, *I*_2_, *I*_3_,…, *I*_112_ using triangular member functions. Output represents the fault location in kilometers which is divided into 111 ranges using triangular member function like *L*_1_, *L*_2_, *L*_3_,…, *L*_111_. Total number of rules designed for series fault location is 112 as given below:

(1) If input is *I*_1_ then location is *L*_1_.
(2) If input is *I*_2_ then location is *L*_2_.
 ⋮
(111) If input is *I*_111_ then location is *L*_111_.
(112) If input is *I*_112_ then location is *L*_1_.

#### 3.3.2. Fuzzy Module for Shunt Fault Location (FIS-LSH)

For designing fault location module for shunt faults using fuzzy inference system, the phase impedance *Z* is calculated for faulty phase(s) from

(6) $$\begin{array}{c} Z=\frac{V_{f}}{I_{f}}, \end{array}$$

where *V*_*f*_ is fundamental component of voltage and *I*_*f*_ is fundamental component of current. *Z* is taken as input to the fuzzy module for fault location estimation of shunt faults. Different fuzzy modules for different types of fault (LG, LLG, LL, and LLL) for fault location estimation are designed. Based on type of fault which has occurred in the system identified by fault classification module, the corresponding FIS-LSH will be activated and fault location will be estimated.  Figure 4(b) shows the fuzzy based fault location modules (FIS-LSH) of different types of shunt fault; impedance (*Z*) of faulty phase is taken as input to fuzzy module and fault location is estimated. In Figure 4(b), it is clear that for LG faults there will be only one input *Z* as only one phase is faulty. Fundamental components of impedance (*Z*) are divided into a number of ranges like *Z*_1_, *Z*_2_, *Z*_3_,…, *Z*_56_ using triangular member functions. Output fault location is also divided into ranges using triangular member function like *L*_1_, *L*_2_, *L*_3_,…, *L*_55_. Total number of rules made for LG shunt fault location is 56. The rules are given below:

(1) If input is *Z*_1_ then location is *L*_1_.
(2) If input is *Z*_2_ then location is *L*_2_.
 ⋮
(55) If input is *Z*_55_ then location is *L*_55_.
(56) If input is *Z*_56_ then location is *L*_1_.

For LLG and LL faults, there are two inputs to the fuzzy module as shown in Figure 4(b). Fundamental components of impedance values for faulty phase 1 (*Z*_i_) and phase 2 (*Z*_ii_) are divided into ranges like *Z*_i1_, *Z*_i2_, *Z*_i3_,…, *Z*_i56_ and *Z*_ii1_, *Z*_ii2_, *Z*_ii3_,…, *Z*_ii56_ using triangular member functions. Output fault location is also divided into ranges using triangular member function like *L*_1_, *L*_2_, *L*_3_,…, *L*_55_. The rules for LLG faults are given hereunder. Similarly LLL fault location module is designed using three inputs for location estimation as shown in Figure 4(b):

(1) If input 1 is *Z*_i1_ and input 2 is *Z*_ii1_ then location is *L*_1_.
(2) If input 1 is *Z*_i2_ and input 2 is *Z*_ii2_ then location is *L*_2_.
(3) If input 1 is *Z*_i3_ and input 2 is *Z*_ii3_ then location is *L*_3_.
 ⋮
(55) If input 1 is *Z*_i55_ and input 2 is *Z*_ii55_ then location is *L*_55_.
(56) If input 1 is *Z*_i56_ and input 2 is *Z*_ii56_ then location is *L*_1_.

#### 3.3.3. Simultaneous Series and Shunt Fault Location

If both series and shunt fault classification FIS modules (FIS-CSH and FIS-CSR) detect the presence of fault, then simultaneous series and shunt fault has occurred. In case of simultaneous series and shunt faults, location of series fault will be obtained using one-end measurement and that of shunt fault will be obtained using remote end measurement. For simultaneous series and shunt faults, FIS-LSR is activated for series fault end and FIS-LSH is activated for shunt fault end, which determines the location of respective fault.

## 4. Results and Discussion

Performance of the proposed method is evaluated by varying different fault parameters like fault type, fault location, and fault inception angle. Fault resistance in case of shunt faults is kept constant (*R*_*f*_ = 0.001 Ω) in proposed method. The FIS module is designed to give output fault location as 500 km during no fault/normal operating condition and during faulty condition; it will give an estimated fault location as output. The percentage of error in fault location estimation is calculated using (7). The test results for different series faults, shunt faults, and simultaneous series and shunt faults are discussed in detail in this section:

(7) $$\begin{array}{c} \text{\% Error}=\frac{\left[\text{Actual Fault Location}-\text{Estimated Fault Location}\right]}{\text{Total Line Length}}*100. \end{array}$$

### 4.1. Series Fault Classification and Location Estimation

**Figure 5:**
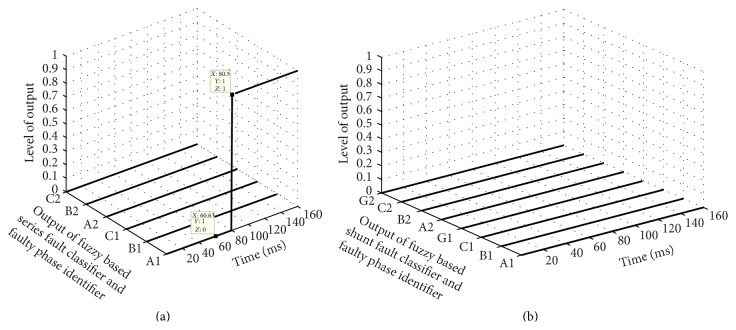
Output of fuzzy based fault classification modules during A1 phase series fault at 64 km in 60 ms time. (a) FIS-CSR and (b) FIS-CSH.

**Figure 6:**
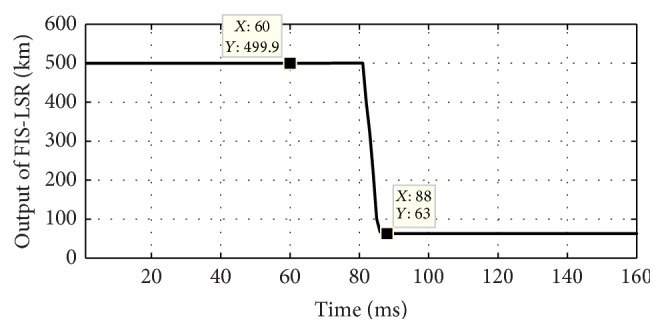
Output of fuzzy based fault location module FIS-LSR during A1 phase series fault at 64 km at 60 ms time.

The proposed scheme involves two stages; first is fault classification and then location estimation. In the first stage, both the FIS modules FIS-CSR and FIS-CSH are tested to detect the fault and classify the fault type, that is, whether the fault is series or shunt fault. For example, a series fault in A1 phase has occurred at 60 ms time and 64 km away from the relaying point; the test result of both the FIS is shown in Figure 5.  Figure 5(a) shows the six outputs of FIS-CSR which become high (1) after 80.5 ms for phase A1 only depicting that the fault is series fault in A1 phase of circuit 1 while Figure 5(b) shows that the outputs of FIS-CSH are all low (0) confirming that there is no shunt fault in the system. Once the fault type is classified as series fault, the corresponding FIS module for series fault location estimation is activated and the output of FIS-LSR during a series fault in phase A1 at 64 km at 60 ms time is shown in Figure 6. The estimated fault location is 63 km after 88 ms time as shown in Figure 6. Further the proposed scheme is also tested for different series faults with varying fault location and inception angle and some of the results of proposed fault location scheme are given in Table 1. The test result shows the high accuracy in determining the fault location with much less % of error.

### 4.2. Shunt Fault Classification and Location Estimation

**Figure 7:**
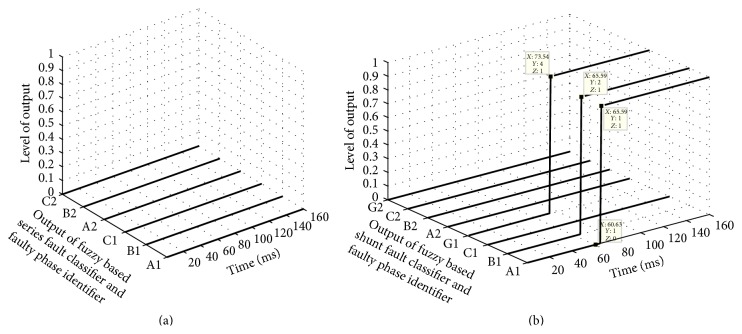
Output of fuzzy based fault classification modules during A1B1G1 shunt fault at 50 km in 60 ms time. (a) FIS-CSR and (b) FIS-CSH.

The proposed scheme can simultaneously detect the presence of fault and also classify the fault whether it is series or shunt fault as, during no fault or normal condition, all outputs of both the fuzzy modules FIS-CSR and FIS-CSH are low (zero), and in case of any type of fault the corresponding fuzzy classification module output changes its state from low to high after some time. This can be clearly seen from the test results shown in Figure 7 during A1B1G shunt fault in circuit 1 at 50 km at 60 ms time.

**Figure 8:**
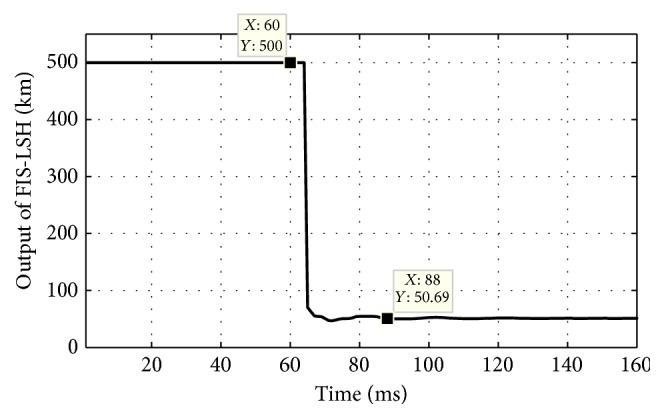
Output of fuzzy based fault location module FIS-LSH during A1B1G fault at 50 km at 60 ms time.

Figure 7(a) shows that all the outputs of FIS-CSR are low throughout the simulation time (0–160 ms) and Figure 7(b) shows the outputs of FIS-CSH fault module which become high for phases A1, B1, and G1 after 73.54 ms time verifying that it is A1B1G shunt fault in circuit 1. As the fault type classified is LLG type of shunt fault, LLG shunt fault locator estimates the fault location as 50.69 km as shown in Figure 8. Few more other types of shunt fault are tested and results are reported in Table 2.

### 4.3. Simultaneous Series and Shunt Faults Location

**Figure 9:**
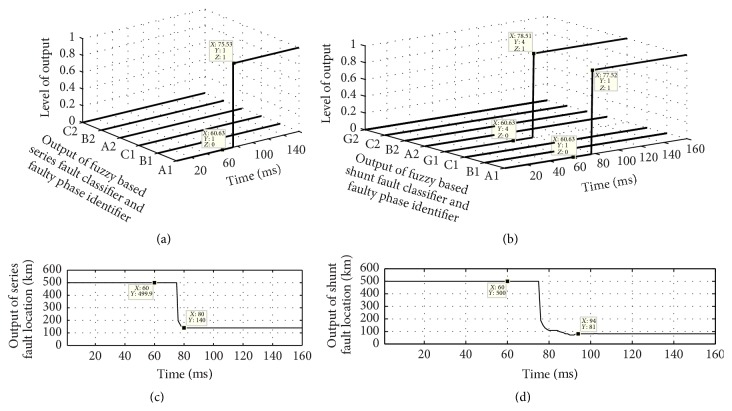
Outputs of fuzzy modules during A1G1 shunt fault at 80 km from sending end at 60 ms time and A1 series fault at 140 km from receiving end. (a) FIS-CSR, (b) FIS-CSH, (c) FIS-LSR, and (d) FIS-LSH.

The simultaneous fault may consist of two different types of fault: series and shunt at the one location or at different locations [27, 28]. For example, consider open circuit in one phase and simultaneously a single phase to ground fault occurring coincidentally on the same phase or different phase at the same location in the transmission line network. A simultaneous series and shunt fault is simulated by assuming that one end of the conductor has been broken and fallen to the ground, while the other end of the conductor is hanged on the tension tower without touching the ground. The proposed method is tested for this situation and Figures 9(a)–9(d) show the test results during A1G shunt fault at 80 km from sending end at 60 ms time and simultaneously the other end of A1 phase conductor is open, that is, A1 phase series fault at the same location from sending end but 140 km from remote end bus. The output of proposed FIS-CSR and FIS-LSR during series fault is shown in Figures 9(a) and 9(c) and Figures 9(b) and 9(d) show the outputs of FIS-CSH and FIS-LSH for shunt fault, respectively. From Figure 9 it can be seen that the proposed scheme correctly identifies the fault type and the faulty phase and its location. Proposed fuzzy based method is tested for some other simultaneous series and shunt faults and results are given in Table 3, which corroborate that the proposed scheme works equally well during simultaneous series and shunt faults situation also, as compared to existing schemes which fails.

### 4.4. Validation of % of Error in Fault Location Using Chi Square (*χ* ^2^) Test

**Figure 10:**
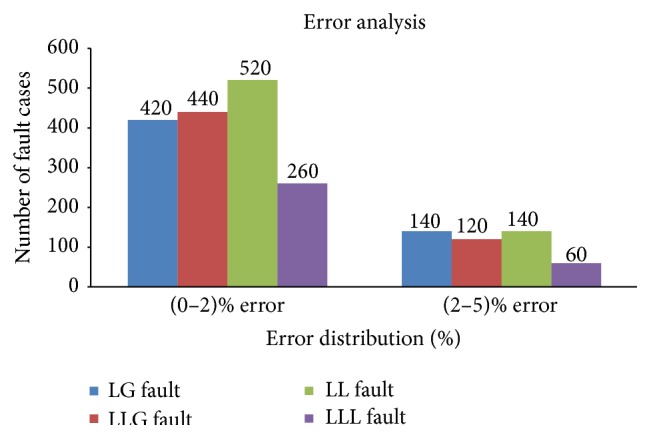
% of error distribution for different faults: LG fault, LLG fault, LL fault, and LLL fault.

A large number of fault case studies had been carried out to evaluate the performance of proposed method by varying different fault parameters like types of fault, fault location, and fault inception angle and % of error in location estimation is calculated. The % of error in series faults location estimation lies within 1%; on the other hand the error lies between 0–5% in case of shunt faults; thus it is necessary to validate it using some validation test, for example, Chi square (*χ* ^2^) test. Fault location error of shunt faults is divided into two ranges 0 to ±2.0% and ±2.1 to ±5%.  Figure 10 shows the percentage of fault cases in which the shunt fault location error is within 0 to ±2.0% and ±2.1 to ±5% ranges.

The computed value of *χ* ^2^ must equal or exceed the appropriate critical value to justify rejection of the null hypothesis at 0.05 or 0.01 level of significance. It shows whether the apparent differences or relationships are true differences/relationships or whether they merely result from sampling error [29]. Chi square test results are shown in Table 4. Fault cases for different range of error according to fault type are the calculated ones which is the observed frequency of error distribution (*f*_*o*_). Expected frequency of occurrence of error for each of the cells (column or row) for all types of fault is calculated from the observed frequency of the error using (8).  *χ* ^2^ is calculated for all types of fault for all the ranges of error and shown in Table 4 by using (9). Different levels of significance for different degrees of freedom are shown in Table 5. Degree of freedom can be calculated as per (10). Consider

(8) $$\begin{array}{c} f_{e}=\left[\frac{\left\{\left(\sum f_{c}\right)*\left(\sum f_{r}\right)\right\}}{\text{Total}}\right], \end{array}$$

where *f*_*e*_ is the expected frequency of error, *f*_*c*_ is observed frequencies in columns, *f*_*r*_ is observed frequencies in rows, and total is the sum of all the frequencies = 2100. Consider

(9) $$\begin{array}{c} \chi^{2}=\sum \left\{\frac{\left(f_{o}-f_{e}\right)}{f_{e}}\right\}, \end{array}$$

where *f*_*o*_ is the observed frequency of error. Consider

(10) $$\begin{array}{c} D=\left(\text{Rows}-\text{1}\right)*\left(\text{Columns}-\text{1}\right), \end{array}$$

where *D* is the degree of freedom.

In this method there are 4 rows and 2 columns, so degree of freedom is 3. From Table 5, with degree of freedom 3, calculated *χ* ^2^ value is less for both 5% and 1% significant levels for all types of fault. This shows that null hypothesis is accepted and the error for fault location will not be the same for replication of experiment. Thus the proposed fuzzy based fault location scheme is accurate and can be used for series and shunt fault location estimation.

## 5. Conclusion

This paper proposes a new approach using synchronized phasor measurement and fuzzy system to classify the series, shunt, and simultaneous series-shunt faults and predict the fault location in a double circuit transmission lines. Proposed method is effective in determining accurate fault location because it is not affected by variation in fault type, fault inception angle, fault distance, and so forth. Fuzzy inference system used for series and shunt fault location estimation is “Mamdani” type. Fault location error in case of series fault is within 1%, while in case of shunt fault it is up to 5%. So error validation of shunt faults is done using Chi square test. The major contribution of the proposed scheme is that it classifies the fault type (both series and shunt) correctly and estimates the correct value of fault location of series faults and simultaneous series-shunt faults which has not been reported to date.

## Figures and Tables

**Table 1:** Test results of series faults location estimation.

Fault type	Fault inception angle (°)	Fault location (km)	Estimated location (km)	Error (%)
A1	0	6	7.000	−0.0045
B2	45	36	35.000	0.0045
C1	90	66	66.000	0.0000
A2B2	135	96	96.000	0.0000
B1C1	180	126	126.000	0.0000
C2A2	225	156	157.000	−0.0045
A1B1C1	270	186	189.000	−0.0136

**Table 2:** Test results of shunt faults location estimation.

Fault type	Fault inception angle (°)	Fault location (km)	Estimated location (km)	Error (%)
A1G	0	9	9.000	0.0000
B2G	45	29	27.000	0.0090
C1G	90	49	49.665	−0.0030
A2B2G	135	69	70.200	−0.0050
B1C1G	180	89	88.640	0.0010
C2A2G	225	109	109.200	−0.0009
A1B1	270	129	132.600	−0.0163
B2C2	315	149	148.200	0.0036
C1A1	0	169	171.200	−0.0100
A2B2C2	90	189	195.000	−0.0272

**Table 3:** Test results of simultaneous series and shunt fault location estimation.

Fault inception angle (°)	Series fault type	Estimated series fault location (km)	Error (%)	Shunt fault type	Estimated shunt fault location (km)	Error (%)
0	A1 at 200 km from receiving end	196.000	1.818	A1G at 20 km from sending end	18.000	0.909
60	B1 at 180 km from receiving end	182.000	0.909	B1G at 40 km from sending end	36.000	1.818
120	C1 at 160 km from receiving end	161.000	0.454	C1G at 60 km from sending end	54.000	2.727
180	A2 at 140 km from receiving end	140.000	0.000	A2G at 80 km from sending end	76.000	1.818
240	B2 at 120 km from receiving end	119.000	0.454	B2G at 100 km from sending end	98.000	0.909
300	C2 at 100 km from receiving end	98.000	0.909	C2G at 120 km from sending end	118.000	0.909
0	C1 at 209 km from receiving end	216.000	−0.0318	A1G at 11 km from sending end	9.000	0.0090
45	B2 at 22 km from sending end	21.000	0.0045	C2G at 198 km from receiving end	199.000	−0.0045
90	A1 at 177 km from receiving end	182.000	−0.0227	B1G at 43 km from sending end	45.000	−0.0090
135	A2B2 at 63 km from sending end	63.000	0	C2G at 157 km from receiving end	165.000	−0.0363
180	A1 at 137 km from receiving end	140.000	−0.0136	B1C1G at 83 km from sending end	85.800	−0.0127
225	B2 at 103 km from sending end	105.000	−0.0090	C2A2G at 117 km from receiving end	117.000	0.0000
270	C1 at 97 km from receiving end	98.000	−0.0045	A1B1 at 123 km from sending end	124.800	−0.0081
315	A2 at 143 km from sending end	147.000	−0.0181	B2C2 at 77 km from receiving end	78.000	−0.0045
0	C1A1 at 57 km from receiving end	56.000	0.0045	B1G at 163 km from sending end	171.000	−0.0363
90	A2B2 at 183 km from sending end	182.000	0.0045	C2G at 37 km from receiving end	36.000	0.00454
180	A2B2C2 at 203 km from sending end	203.000	0	A2G at 17 km from receiving end	18.000	−0.0045
270	A1B1 at 213 km from sending end	215.000	−0.0090	B1G at 7 km from receiving end	9.000	−0.0090

**Table 4:** *χ* ^2^ test for fault location error for different types of fault.

Fault type	Parameters	Range of percentage of error		∑
		0% to ±2%	±2% to ±5%	
LG	*f*_*o*_	420	140	$\sum \left\{\frac{{\left(f_{o}-f_{e}\right)}^{2}}{f_{e}}\right\}=3.315$
	*f*_*e*_	437.333	122.666	
	(*f*_*o*_ − *f*_*e*_)^2^/*f*_*e*_	0.686	2.449	

LLG	*f*_*o*_	440	120	$\sum \left\{\frac{{\left(f_{o}-f_{e}\right)}^{2}}{f_{e}}\right\}=0.073$
	*f*_*e*_	437.333	122.666	
	(*f*_*o*_ − *f*_*e*_)^2^/*f*_*e*_	0.016	0.057	

LL	*f*_*o*_	520	140	$\sum \left\{\frac{{\left(f_{o}-f_{e}\right)}^{2}}{f_{e}}\right\}=0.188$
	*f*_*e*_	515.428	144.571	
	(*f*_*o*_ − *f*_*e*_)^2^/*f*_*e*_	0.040	0.148	

LLL	*f*_*o*_	260	60	$\sum \left\{\frac{{\left(f_{o}-f_{e}\right)}^{2}}{f_{e}}\right\}=0.9092$
	*f*_*e*_	249.904	70.095	
	(*f*_*o*_ − *f*_*e*_)^2^/*f*_*e*_	0.407	1.453	

$\chi^{2}[all]=\sum \left\{\frac{{\left(f_{o}-f_{e}\right)}^{2}}{f_{e}}\right\}=4.485$				

**Table 5:** Levels of significance for different degrees of freedom.

Degree of freedom	Level of significance	
	5%	1%
1	3.84	6.64
2	5.59	9.21
3	7.82	11.34
4	9.49	13.28
5	11.07	15.09
